# Comparison of outcomes between unilateral biportal endoscopic discectomy and percutaneous transforaminal endoscopic discectomy in single-level lumbar disc herniation

**DOI:** 10.3389/fsurg.2026.1863660

**Published:** 2026-07-29

**Authors:** Junyang Li, Jianjian Yin, Tao Ma, Jingwei Wu, Xiaoshuang Tu, Luming Nong, Gongming Gao

**Affiliations:** 1 Orthopedics Department, The Third Affiliated Hospital of Nanjing Medical University, Changzhou, Jiangsu Province, China; 2 Department of Orthopedics, Nanjing Medical University, Nanjing, Jiangsu Province, China; 3 Changzhou Medical Center, Nanjing Medical University, Changzhou, Jiangsu Province, China

**Keywords:** full-endoscopic, lumbar disc herniation, minimally invasive, percutaneous transforaminal endoscopic discectomy, unilateral biportal endoscopic discectomy

## Abstract

**Purpose:**

The clinical results of percutaneous transforaminal endoscopic discectomy (PTED) and unilateral biportal endoscopic discectomy (UBED) for the treatment of L4/L5 disc herniation were compared.

**Methods:**

Patients with L4/L5 disc herniation who had either Percutaneous Transforaminal Endoscopic Discectomy (PTED; *n* = 53) or Unilateral Biportal Endoscopic Discectomy (UBED; *n* = 69) at our facility during the same time period were the subject of a retrospective evaluation. Clinical outcomes, radiological information, and complications were collected and evaluated for each group.

**Results:**

A retrospective analysis of 122 patients with L4/L5 disc herniation revealed that 69 of them received unilateral biportal endoscopic discectomy (UBED) and 53 got percutaneous transforaminal endoscopic discectomy (PTED). Leg pain (*P* = 0.242) and low back pain (*P* = 0.645) preoperative, postoperative day 1, and final follow-up VAS scores did not show any significant between-group differences. By the final follow-up, Oswestry Disability Index (ODI) scores were significantly improved for both groups (*P* < 0.001). The UBED group's patient satisfaction rate was 95.7%, while the PTED group's was 94.0%, according to the modified MacNab criteria. The two groups did not differ statistically significantly (*P* = 1). Similarly, radiological outcomes did not differ significantly between groups. Compared to the PTED group, patients in the UBED group experienced higher hemoglobin loss and longer hospital stays. On the other hand, intraoperative fluoroscopy exposures were dramatically decreased (*P* < 0.01) by the UBED method.

**Conclusion:**

Both UBED and PTED are effective and safe treatment options for L4/L5 disc herniation. UBED entailed less fluoroscopic exposure, and PTED contributed to less blood loss as well as a shorter hospital stay.

## Introduction

Microendoscopic discectomy (MED) surgery (1) is considered a classic technique and the gold standard for treating lumbar disc herniation (LDH). But in recent years, both surgeons and patients have increasingly pursued smaller tissue injuries and faster postoperative recovery. The percutaneous transforaminal endoscopic discectomy (PTED) technique, which establishes a channel through puncture of the Kambin triangle and removes the nucleus pulposus tissue under endoscopy, has been widely performed and proven to be as effective as MED in long-term follow-up studies (2). However, PTED has a high learning curve in contrast to the anatomical nature of traditional posterior surgery, where the working range is constrained by the working channel.

The unilateral biportal endoscopic (UBE) approach, which originated from arthroscopic surgery (3), allows surgeons to handle surgical tools more effectively and flexibly through working channels and observation to accomplish a greater range of decompression (4). UBE discectomy is comparable to traditional MED in terms of pain relief and functional outcomes. In addition, it has significant advantages in short-term postoperative back pain scoring (5). Study (6) has shown that LDH can be effectively treated by UBE surgery, which has a low risk of complications. However, there is still a minimum learning curve of 54 cases needed to become proficient in UBE surgery.

One is the single-portal endoscopic technique via lateral approach, and the other is the bi-portal endoscopic technique via posterior approach, both of which have wide applications in clinical. This retrospective study aims to investigate the clinical efficacy of the UBED technique in the treatment of L4/5 LDH by comparing with the PTED technique.

### Patients

**Figure 1:**
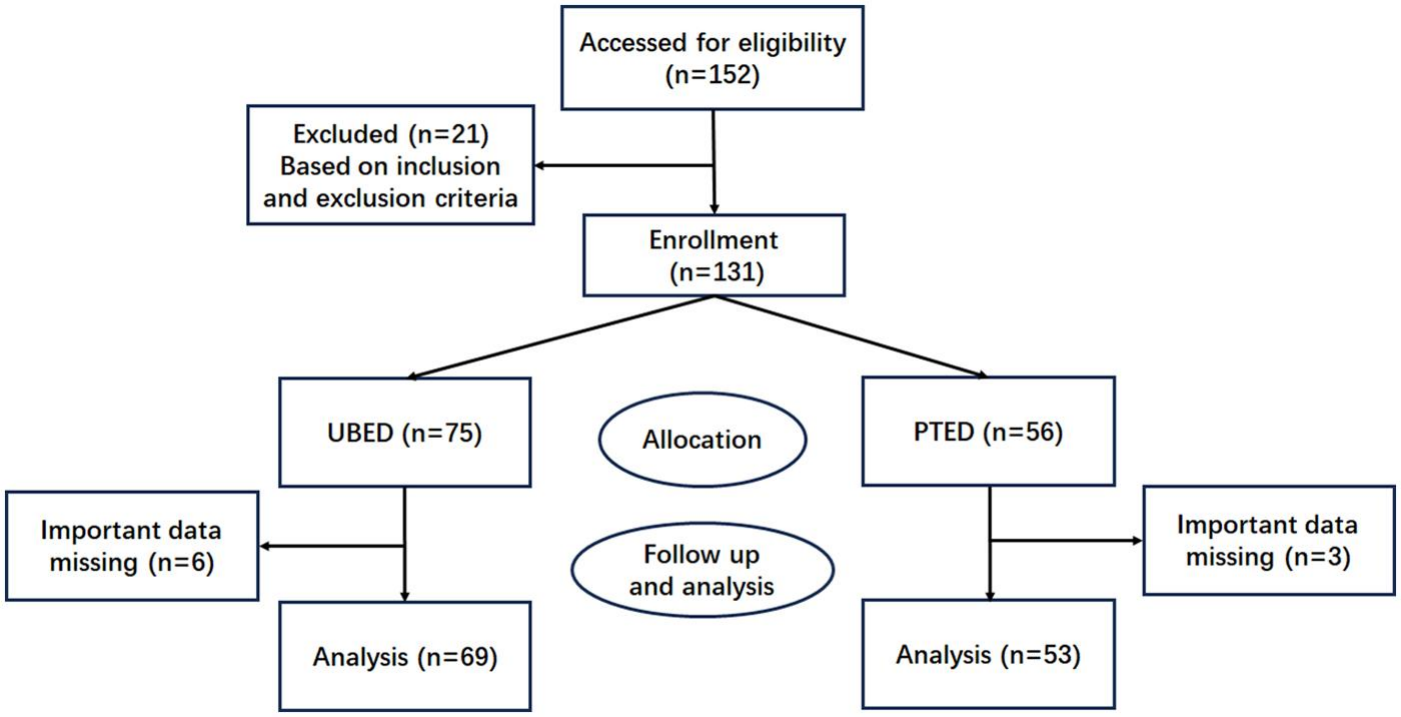
Flow diagram showing the inclusion and exclusion of patients recruited in the study.

A retrospective analysis was conducted on a group of consecutive patients who had L4/5 LDH endoscopic surgery at our hospital between July 2022 and December 2023 (Figure 1).

Inclusion criteria were: (1) back or radiation pain related to L4/5 LDH; (2) magnetic resonance imaging is consistent with symptoms and signs; (3) symptoms persist for more than 6 weeks;

Exclusion criteria were therefore defined: (1) recurrent LDH; (2) extraforaminal disc involvement; (3) degenerative lumbar spinal stenosis; (4) lumbar instability; (5) comorbid tumorous or infectious disease; (6) without complete clinical and radiological data;

### Surgical procedures

All procedures were performed by the same senior surgical team, who had already passed the learning curves for both PTED and UBED before patient enrollment. Thus, all included cases were treated during the stable proficiency stage, minimizing performance bias.

### UBED

**Figure 2:**
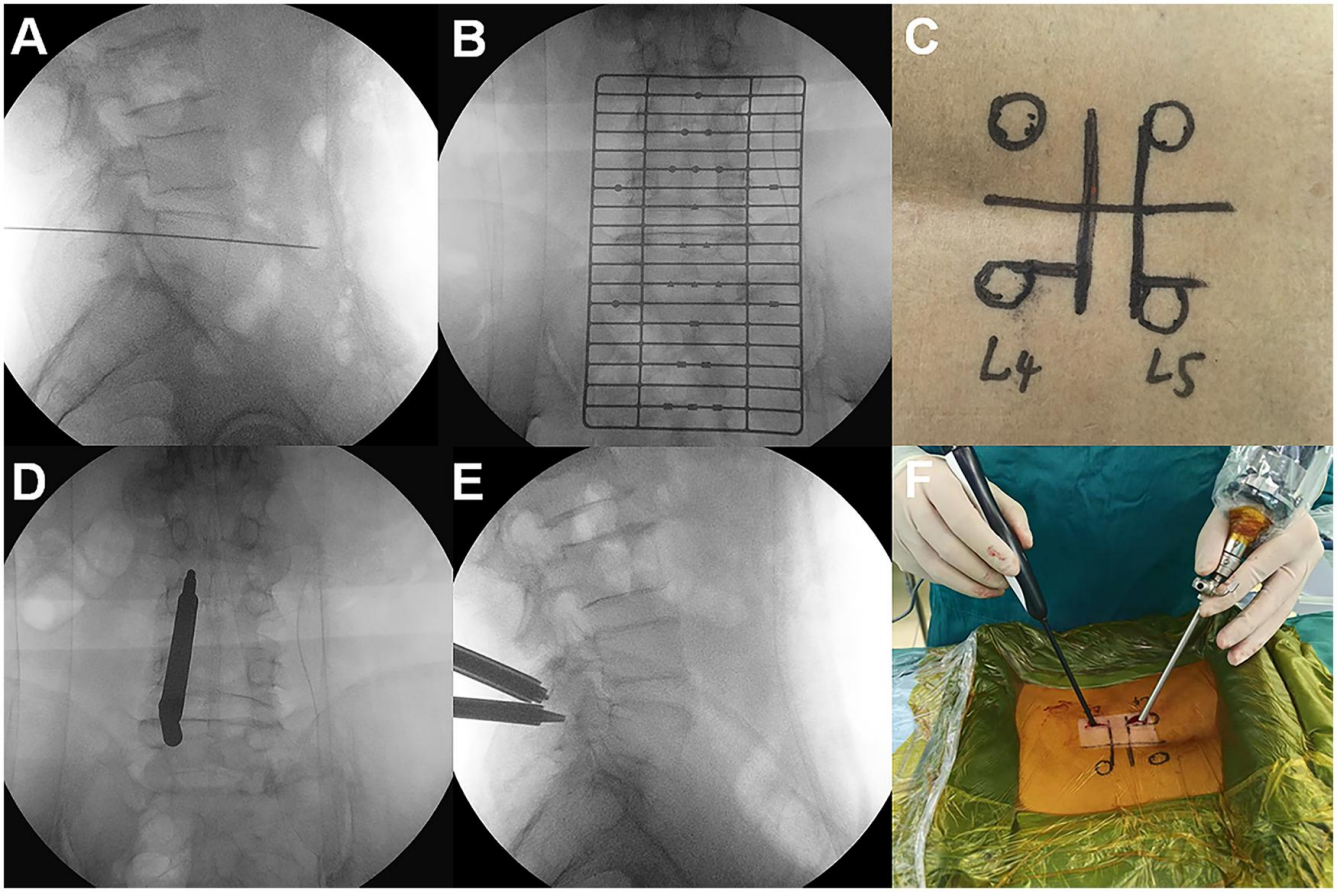
Preparation and safety measures for UBED. **(A)** To ensure that the surgical level is perpendicular to the ground, a Kirschner needle is employed in a lumbar lateral x-ray. **(B)** Using a grid-shaped metal marker to mark the anterior and posterior x-ray film; **(C)** Marking the surface projection of the surgical level, pedicles, and midline of the spinous process; **(D,E)** Reconfirming the surgical level using fluoroscopy following skin incision and puncture; **(F)** Using both hands to simultaneously operate the electrical coagulator and endoscope during surgery.

UBED was conducted under general anaesthesia (7). The surgical table was adjusted to cause slight lumbar flexion and increase intervertebral space while the patient was in a prone position. To ensure that the surgical segment is perpendicular to the ground, a standard lumbar lateral x-ray was taken. A grid shaped metal marker was used to mark the surgical segment, pedicles, and spinous process midline according to the lumbar anterior and posterior x-ray images. Two longitudinal incisions of approximately 1 cm along the inner edge of the L4 and L5 pedicle projections were made for endoscopic observation and instrument operation, respectively. The sleeve gradually expands and bluntly separates to the bony surface. In this step, it was necessary to confirm the surgical segment again by fluoroscopy of the lumbar anteroposterior and lateral radiographs (Figure 2).

**Figure 3:**
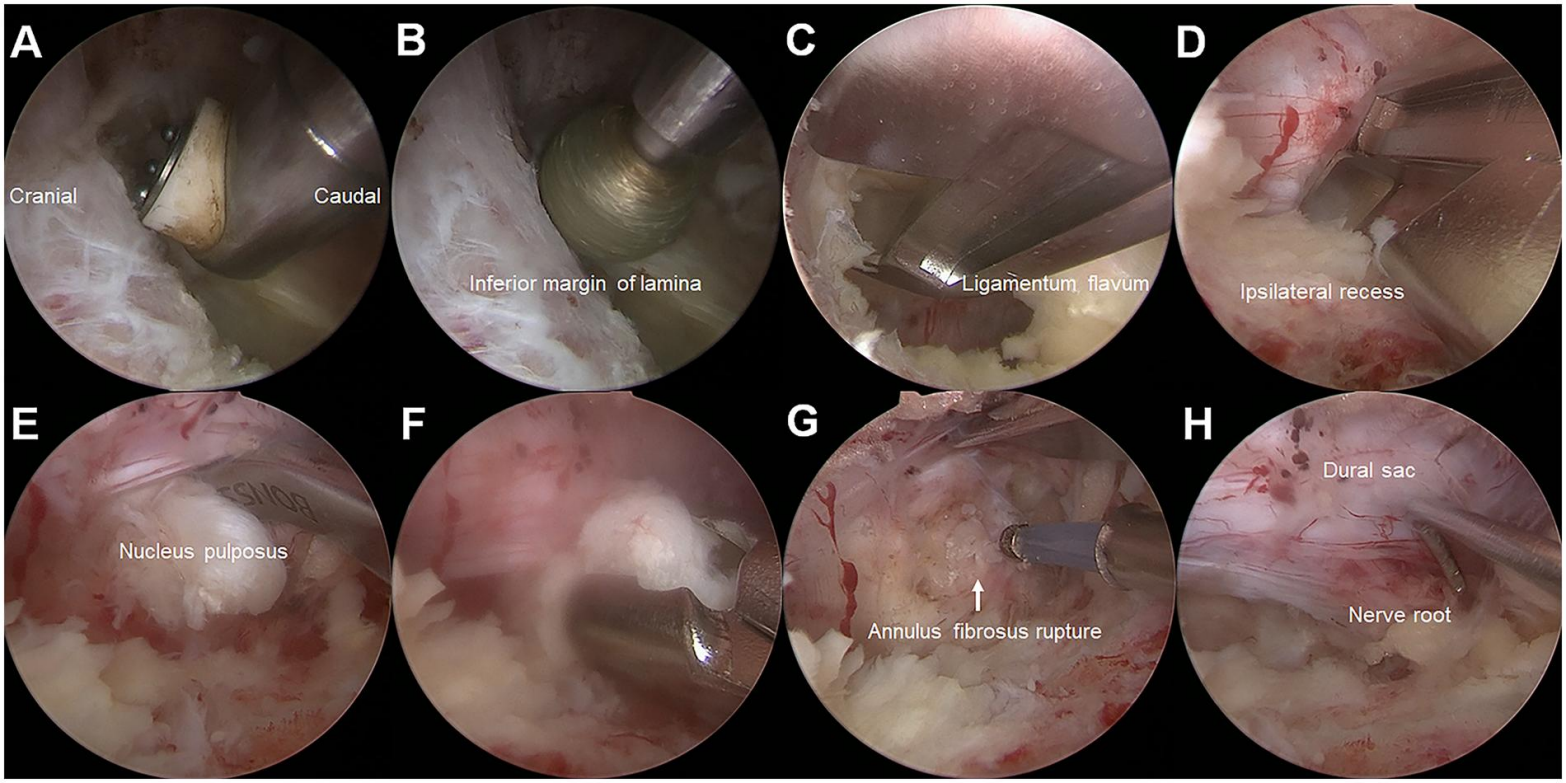
UBED for herniation of the L4/L5 disc. **(A)** An electrical coagulator was used to separate muscle from bone; **(B)** A high-speed microdrill was used to resect the ipsilateral laminotomy; **(C)** Make the ligamentum flavum visible and remove it; **(D)** decompression of the ipsilateral recess; **(E)** In the axillary position, reveal the herniated nucleus pulposus (arrow indicated); **(F)** Excise the herniated nucleus pulposus; **(G)** The disc annulus fibrosus rupture arrow; **(H)** Sufficient decompression is indicated by the relaxation of the dural sac and nerve roots.

The lower edge of the L4 lamina and the interlaminar gap were determined following the separation of muscle and bone using an electrical coagulator. Lamina fenestration was performed with high-speed microdrill. Before performing flavectomy with the Kerrison punch, a neural probing hook was used to break through the ligamentum flavum. Appropriate ipsilateral recess decompression was necessary to create sufficient room for shoulder movement since the nucleus pulposus protrusion was pressing against neural structures. Gently pulling the nerve inward through the neural hook can help expose and remove the nucleus pulposus. Meanwhile, further exploration of the axillary area was needed to ensure adequate nerve root decompression (Figure 3).

### PTED

The PTED procedure has been described previously (7). The surgery was performed using local anesthesia combined with intravenous sedation, and the patient remained conscious during the procedure. C-arm fluoroscopy is used to determine the surgical segment, with the puncture point located approximately 11–14 cm from the midline of the intervertebral space plane.

**Figure 4:**
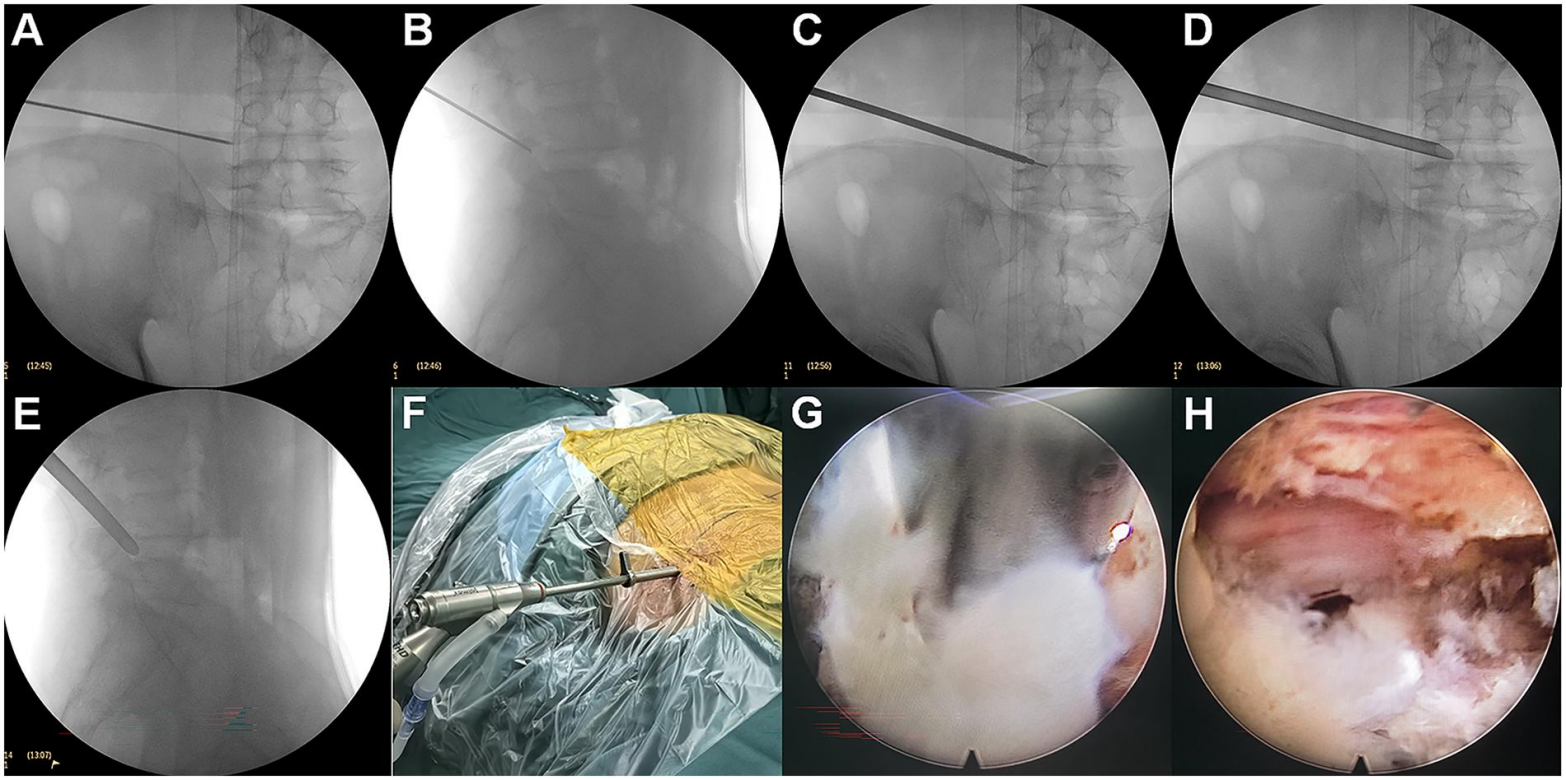
PTED for herniation of the L4/L5 disc. **(A,B)** The puncture needle reaches the ventral aspect of the L5 superior articular process; **(C)** Sequential dilation of the soft tissue is performed using sleeves, followed by endoscopic device system with a drill; **(D,E)** The working cannula is placed; **(F)** The endoscopic system is connected; **(G)** The nucleus pulposus tissue is removed; **(H)** Nerve root relaxation is confirmed upon exploration.

After the puncture needle tip reaches the ventral side of the L5 superior facet joint, the sleeve gradually expands the soft tissue and uses a grinding drill to polish the facet joint and enlarge the intervertebral foramen. When the lateral view of the puncture needle reaches the posterior edge of the intervertebral disc and the anteroposterior view exceeds the inner edge of the pedicle, a working channel can be inserted. Due to the limitations of single hole endoscopy, it is necessary to adjust and rotate the working channel appropriately in order to obtain better surgical operating space. Soft tissues such as the ligamentum flavum, nerve roots, posterior longitudinal ligament, and nucleus pulposus need to be carefully identified from top to bottom. After the protruding nucleus pulposus is removed, the nerve roots can freely pulsate with water pressure (Figure 4).

### Clinical measurements

From the preoperative phase until the final follow-up, all data was gathered. Pain intensity, quality of life, and patient satisfaction were measured using the Visual Analog Scale (VAS) for back and leg pain, the Oswestry Disability Index (ODI) score, and the Modified Macnab Scale (excellent, good, fair, and poor).

General information, including age, gender, and body-mass index (BMI), was assessed based on the patients’ prior medical records. The Michigan State University (MSU) classification method was used to identify the type and severity of LDH (7). Perioperative data, such as the length of the procedure, the length of the hospital stay after surgery, and any complications, has been obtained from the patient's hospitalization medical record. Hemoglobin (Hb) and hematocrit (Hct) were measured the day before and after surgery.

### Imaging measurements

Lumbar anteroposterior and lateral x-rays, as well as lumbar extension and flexion x-rays, were obtained for both patient groups prior to, during, and following surgery. Additionally, magnetic resonance imaging (MRI) and lumbar CT scans were only performed before to surgery. Two independent evaluators measured and averaged all imaging data.

To determine the intervertebral height, we employ the modified distortion compensated Roentgen analysis (DCRA) (8). Intervertebral height was determined by averaging the anterior, middle, and posterior distances from the upper and lower vertebral bodies to the midline on a lateral radiographic view. Intervertebral height index (IHI) was established as the ratio of intervertebral height to anterior and posterior diameter of the upper vertebral body (9).

Range of motion (ROM) is calculated using the segmental angle on the lateral radiographs in both extended and flexed positions. The segmental angle is the angle formed by the line connecting the inferior endplate of the upper vertebra and the superior endplate of the lower vertebra. The difference between the segmental angles in flexion and extension is known as the segmental range of motion (Figure 3).

### Statistical analysis

Statistical analyses were performed using SPSS version 22.0 (IBM Corp., Chicago, IL, USA). A *P*-value < 0.05 was considered statistically significant. Continuous variables were presented as mean ± standard deviation (SD) for normally distributed data, and as median (interquartile range, IQR) for non-normally distributed data. Between-group comparisons were performed using the independent t-test or Mann–Whitney U test, while within-group pre–post comparisons were analyzed using the paired t-test. Repeated measurements at different time points were analyzed using repeated-measures analysis of variance (ANOVA). Categorical variables were compared using the Chi-square test or Fisher's exact test.

## Result

### Patient population

A review was conducted on 152 patients undergoing LDH surgery. 21 patients were initially disqualified because they did not fit the inclusion and exclusion criteria. Nine more patients were eliminated from the data collection procedure as a result of missing crucial information and missed follow-up. In the end, 122 patients were found in the trial; 53 individuals were included in the PTED group and 69 patients made up the UBED group. The flow diagram is shown in Figure 1.

A total of sixty-six (54.1%) males and fifty-six (45.9%) females were included in our study. Their mean age were 55.35 ± 13.92 years and 55.40 ± 16.55 years, respectively. The follow-up time for the two groups were 22.00 (17.00, 26.00) and 23.00 (19.00, 26.00) months.

No significant differences were observed in the basic characteristics, including age, sex, and MSU Classification, between the two groups (Table 1).

**Table 1:** Demographic and clinical characteristics for all the patients.

Index	PTED (*n* = 53)	UBED (*n* = 69)	*P*-value
Age (years)	55.40 ± 16.55	55.35 ± 13.92	0.986
BMI (kg/㎡)	23.85 ± 2.92	24.61 ± 3.20	0.180
Male/Female (n)	32/21	34/35	0.223
Hypertension (n)	22	25	0.553
Diabetes mellitus (n)	10	6	0.099
MSU Classification(n)			0.986
2A	11	14	
2AB	14	17	
2B	21	25	
3A	2	4	
3AB	2	3	
3B	3	6	
Fluoroscopic times (n)	17.00 (15.00, 22.00)	5.00 (5.00, 5.00)	<0.001
Operation time (min)	70.00 (60.00, 95.00)	75.00 (55.00, 100.00)	0.616
Hospital stay after operation (day)	2.00 (2.00, 3.00)	3.00 (2.00, 5.00)	<0.001
Follow up time (months)	23.00 (19.00, 26.00)	22.00 (17.00, 26.00)	0.352
Preoperative Hb (g/L)	139.58 ± 18.89	141.54 ± 14.47	0.519
Postoperative Hb (g/L)	129.34 ± 18.08	127.91 ± 12.44	0.624
Hb loss, g/L (g/L)	−8.00(−17.00, −5.00)	−15.00(−18.00, −8.00)	0.026
Preoperative Hct (%)	40.87 ± 4.77	41.40 ± 4.11	0.505
Postoperative Hct (%)	37.81 ± 4.67	37.45 ± 3.52	0.640
Hct loss (%)	−3.05 ± 2.29	−3.95 ± 2.85	0.063
Preoperative DHI (%)	0.28 ± 0.08	0.30 ± 0.06	0.172
Postoperative DHI (%)	0.27 ± 0.07	0.28 ± 0.06	0.202
Preoperative ROM (°)	8.30 (5.98, 9.02)	8.44 (7.13, 9.24)	0.338
Postoperative ROM (°)	8.78 (7.80, 9.32)	8.58 (7.57, 9.41)	0.901
Complications (n)	3	3	1.000
Nerve root injury (n)	2	1	
Dural tear (n)	0	1	
Nucleus pulposus residue (n)	1	1	
Modified MacNab evaluation (excellent/good/fair/poor)	28/22/3/0	36/30/3/0	1.000
Excellent/good rate (%)	94	95.70	

### Clinical outcome

The operation time between the two groups were similar [75.00 (55.00, 100.00) and 70.00 (60.00, 95.00) min] (*P* = 0.616). The hospital stay time after operation was shorter in the PTED group [2.00 (2.00, 3.00) days] compared with UBED group [3.00 (2.00, 5.00) days] (*P* < 0.001). PTED group received more fluoroscopy sessions than UBED group [17.00 (15.00, 22.00) vs 5.00 (5.00, 5.00)] (*P* < 0.001).

Post-op Hb and Post-op Hct were 127.91 ± 12.44 g/L, 37.45 ± 3.52% in UBED group and 129.34 ± 18.08 g/L, 37.81 ± 4.67%, respectively. No difference was found between two groups (*P* = 0.624) (*P* = 0.640). The Hb loss of UBED group was more than that in PTED group **(**Table 1**)**.

In the UBED group and PTED groups, VAS leg and lumbar scores were significantly improved after surgery and the differences were statistically significant (Table 2). One day postoperatively, the VAS leg and lumbar scores in the PTED group decreased from preoperative values of 6.58 ± 0.93 and 5.11 ± 0.85 to 2.19 ± 0.94 and 2.25 ± 0.52, respectively; in the UBED group, these scores decreased from 7.09 ± 0.97 and 5.57 ± 0.98 to 2.36 ± 1.26 and 2.42 ± 0.55, respectively. With the passage of time, the VAS leg and lumbar scores gradually decrease. And there were statistically significant differences between pairwise comparisons within the same group (*P* < 0.001) (*P* < 0.001). However, the VAS leg (*P* = 0.242) and lumbar (*P* = 0.645) scores of the two groups at each time point did not show any statistically significant difference.

**Table 2:** Comparisons of clinical outcomes between the two groups.

	PTED (*n* = 53)	UBED (*n* = 69)	*P* value
VAS leg score			
Preoperation	6.58 ± 0.93	7.09 ± 0.97	Ptime < 0.001, Pgroup < 0.001, Ptime*Pgroup=0.232
Postoperative 1 d	2.19 ± 0.94	2.36 ± 1.26	
Postoperative 3 M	1.36 ± 0.79	1.39 ± 0.83	
Final follow-up	0.57 ± 0.54	0.70 ± 0.81	
VAS lumbar score			
Preoperation	5.11 ± 0.85	5.57 ± 0.98	Ptime < 0.001, Pgroup=0.068, Ptime*Pgroup=0.115
Postoperative 1 d	2.25 ± 0.52	2.42 ± 0.55	
Postoperative 3 M	1.53 ± 0.50	1.72 ± 0.48	
Final follow-up	0.81 ± 0.40	1.09 ± 0.48	
ODI			
Preoperation	67.13 ± 7.17	62.65 ± 6.23	Ptime < 0.001, Pgroup=0.108, Ptime*Pgroup < 0.001
Postoperative 3 M	22.42 ± 5.22	23.61 ± 5.04	
Final follow-up	14.87 ± 4.38	14.67 ± 5.42	

Data are presented as mean ± SD. Repeated measures ANOVA was used for longitudinal comparisons, including the main effects of time and group, as well as their interaction. Ptime represents the within-subject effect over time, Pgroup represents the between-group effect, and Ptime* Pgroup represents the interaction effect. *post hoc* pairwise comparisons were adjusted for multiple testing.

Both groups demonstrated significant improvement in ODI scores at three months postoperatively compared with baseline (statistically significant), with further reduction by the final follow-up. Although the scores changed over time, there was no significant difference between the two groups (*P* = 0.108) (Table 2).

Complications occurred in 3/69 (4.3%) patients in the UBED group and 3/53 (5.7%) patients in the PTED group (*p* = 1). Specifically, in the UBED group, one case each of nerve injury (1.4%), residual nucleus pulposus (1.4%), and dural tear (1.4%) was recorded. In the PTED group, two cases of nerve injury (3.4%) and one case of residual nucleus pulposus (1.7%) were observed, whereas no dural tear occurred in this group. All three patients with nerve injury presented with mild ipsilateral radiating pain, and complete pain relief was achieved following conservative treatments including dehydration therapy. Other patients were resolved via conservative treatment, and no permanent neurological sequelae were noted in any patient.

The satisfaction rates in the UBED and PTED groups were 95.7% and 94%, respectively, according to MacNab criteria, and there was no discernible difference between the two groups (*P* = 1).

### Radiologic measurements

The Pre-op DHI and Pre-op ROM were similar between two groups (Table 1). Post-op DHI were 0.28 ± 0.06 in UBED group and 0.27 ± 0.07 in PTED group, Post-op ROM were 8.58 (7.57, 9.41) in UBED group and 8.78 (7.80, 9.32) in PTED group. No significant difference was found between two groups (*P* = 0.901, *P* = 0.202).

## Discussion

This study conducted a comparative analysis of the clinical outcomes between UBED and PTED in patients with L4/L5 disc herniation. UBED is performed from the dorsal side of neural tissue for decompression, while PTED is performed from the ventral side. According to the published research (10–12), no significant difference was found in postoperative VAS, ODI scores and modified MacNab criteria between the two groups. Similarly, our results show a significant improvement in VAS and ODI scores after surgery for both surgical procedures, with no significant difference observed. And modified MacNab evaluation of both surgical groups indicate a high level of satisfaction. However, due to its technical complexity and the meticulous nature of the surgical procedure, UBE usually requires more complex anesthesia methods and a longer operation duration. These might be the considerations that spine surgeons take into account when choosing a surgical procedure.

The relief of lower limb neurological symptoms often indicates sufficient and effective decompression. In Ding H et al’ s study (13), the average increase in dural sac area in the PTED group was 43.16 ± 14.62 cm^2^, while in the UBED group it was 68.53 ± 16.42 cm^2^. UBE technology can obtain better decompression spaces. Yin J et al. (7) confirmed UBE surgery is also a safe and effective method for treating lumbar spinal stenosis that can significantly improve clinical functional score and CT cross-sectional spinal canal area. For the same amount of nucleus pulposus removal in L4/5, UBED requires a certain degree of laminectomy and ligamentum flavum resection before entering the spinal canal, which may be the reason for the increased cross-sectional area of the dura sac. PTED surgery usually requires shaping the ventral aspect of the superior articular process and enlarging the anterior posterior diameter of the intervertebral foramen to facilitate better insertion of the surgical channel. Both methods can yield relatively satisfactory results.

Chang H et al. confirmed (5) that PTED and UBED can reduce immediate postoperative back pain compared with MED surgery. And we found no significant difference in back pain scores relief between the two surgical methods immediately after surgery and at the last follow-up. People with low back pain (14) have slightly smaller multifidus muscles and a large amount of fat infiltration within the muscles. Research (15) has shown that single-portal and bi-portal surgeries do not have a significant effect on the degree of multifidus atrophy and fat infiltration in patients under 65 years old. However, another study (13) suggests that The UBED group's paraspinal muscle area was considerably larger after surgery and lower a year later compared to that of the PTED group, indicating that the bi-portal procedure may result in more paraspinal muscle atrophy and injury. PTED establishes a working channel in the muscle gap by gradually expanding the sleeve, while UBED often requires stripping the muscle stop on the surface of the vertebral plate to establish a working cavity. There is still controversy over the size of paraspinal muscle injuries during endoscopic surgery, and further large-scale studies are needed.

The ROM is mostly affected by disc and facet joint and is related with stress on the corresponding disc (16). Some studies (16, 17) have indicated that preserved disc height is inversely correlated with lumbar range of motion (ROM), which in turn is associated with lumbar stability. As is well known, discectomy can lead to a decrease in DHI. Furthermore, the decrease in DHI is closely related to the reduction in pain, which explains the decreased postoperative low back pain score (18). Our data indicate comparable outcomes for UBED and PTED in preoperative and postoperative ROM and DHI. This lack of significant difference is consistent with the previous studies suggesting that both techniques are equally effective in upholding spinal stability.

A retrospective study (19) has shown that the length of hospital stay and recovery period for patients with UBED is longer compared to those with PTED. Our research also proved this point. This is because what PTED requires is merely local anesthesia, rather than general anesthesia as is needed in the UBED procedure. Although this difference could theoretically influence perioperative outcomes, two lines of evidence suggest that it does not meaningfully confound our conclusions. Firstly, all anesthetic agents used in both protocols—propofol, sevoflurane, and lidocaine—have short elimination half-lives (ranging from minutes to ∼100 min) and are rapidly cleared from the body, resulting in negligible plasma concentrations by postoperative day 1 (∼24 h after surgery) (20, 21). Secondly, a meta-analysis by Han et al. (22). including 549 patients directly compared local versus general anesthesia in percutaneous interlaminar endoscopic discectomy (PIED) and found no significant differences between the two groups in postoperative therapeutic indicators. This high-level evidence confirms that anesthesia type does not alter the core functional and pain outcomes of endoscopic spine surgery. Therefore, although the anesthesia protocols differed between the UBED and PTED groups, this difference is unlikely to have confounded our primary outcomes, which were assessed starting from postoperative day 1.

In addition, our study demonstrated a greater Hct loss in the UBED group compared to the PTED group. This finding is consistent with reports (23) of higher intraoperative blood loss in UBED, which is attributed to the need for blunt dissection of the multifidus and pectoralis major muscles to manually create an endoscopic working space. Evidence from Wen et al. (24) demonstrates a positive correlation between significant intraoperative blood loss and the incidence of postoperative complications. Accordingly, efforts in spinal surgery should be directed toward reducing perioperative blood loss as a primary means of mitigating surgical risks.

Although improving proficiency in PTED can shorten fluoroscopy duration (25), its application remains unavoidable during key maneuvers: initial puncture, graded soft-tissue dilation, foraminoplasty, and working cannula placement—each of which requires visual confirmation. UBED, however, limits fluoroscopy to segmental localization. Repeated fluoroscopic exposure poses risks of iatrogenic injury to blood vessels, nerves, and internal organs (26), as well as the potential for serious conditions from prolonged radiation, such as cataracts, leukemia, skin erythema, and thyroid canc (27, 28).

## Limitations

There are several limitations to this study that should be acknowledged. First, it was constrained by a brief follow-up period and a relatively small sample size. Second, as a single-center retrospective study, our analysis relied on existing clinical data. Consequently, the generalizability of our findings may be limited by the specific surgical protocols and patient population of this center. Third, we did not perform routine postoperative MRI evaluation, which limits our ability to assess radiographic outcomes or structural changes after surgery. Future prospective, multi-center studies with longer follow-up and standardized imaging protocols are needed to provide more conclusive evidence.

## Conclusion

Both UBED and PTED are effective and safe treatment options for L4/L5 disc herniation. UBED entailed less fluoroscopic exposure, and PTED contributed to less blood loss as well as a shorter hospital stay.

## Data Availability

The original contributions presented in the study are included in the article/Supplementary Material, further inquiries can be directed to the corresponding authors.
